# Applying a private sector capitation model to the management of type 2 diabetes in the South African public sector: a cost-effectiveness analysis

**DOI:** 10.1186/1472-6963-14-444

**Published:** 2014-09-30

**Authors:** Heinrich C Volmink, Melanie Y Bertram, Ruxana Jina, Alisha N Wade, Karen J Hofman

**Affiliations:** Gauteng Department of Health, Chris Hani Baragwanath Academic Hospital, Johannesburg, South Africa; Department of Community Health of the School of Public Health, Faculty of Health Sciences, University of the Witwatersrand, Johannesburg, South Africa; Priority Cost Effective Lessons for Systems Strengthening South Africa (PRICELESS SA), MRC/WITS Rural Public Health and Health Transitions Research Unit, Johannesburg, South Africa; School of Public Health, Faculty of Health Sciences, University of the Witwatersrand, Johannesburg, South Africa; Gauteng Department of Health, Charlotte Maxeke Johannesburg Academic Hospital, Johannesburg, South Africa

**Keywords:** Capitation, Cost-effectiveness, Diabetes mellitus, National health insurance

## Abstract

**Background:**

Diabetes mellitus contributes substantially to the non-communicable disease burden in South Africa. The proposed National Health Insurance system provides an opportunity to consider the development of a cost-effective capitation model of care for patients with type 2 diabetes. The objective of the study was to determine the potential cost-effectiveness of adapting a private sector diabetes management programme (DMP) to the South African public sector.

**Methods:**

Cost-effectiveness analysis was undertaken with a public sector model of the DMP as the intervention and a usual practice model as the comparator. Probabilistic modelling was utilized for incremental cost-effectiveness ratio analysis with life years gained selected as the outcome. Secondary data were used to design the model while cost information was obtained from various sources, taking into account public sector billing.

**Results:**

Modelling found an incremental cost-effectiveness ratio (ICER) of ZAR 8 356 (USD 1018) per life year gained (LYG) for the DMP against the usual practice model. This fell substantially below the Willingness-to-Pay threshold with bootstrapping analysis. Furthermore, a national implementation of the intervention could potentially result in an estimated cumulative gain of 96 997 years of life (95% CI 71 073 years – 113 994 years).

**Conclusions:**

Probabilistic modelling found the capitation intervention to be cost-effective, with an ICER of ZAR 8 356 (USD 1018) per LYG. Piloting the service within the public sector is recommended as an initial step, as this would provide data for more accurate economic evaluation, and would also allow for qualitative analysis of the programme.

## Background

South Africa is undergoing an epidemiological transition characterized by a growing burden of non-communicable diseases [1]. Diabetes mellitus features prominently within this group. The prevalence of diabetes (type 1 and type 2) amongst adults (ages 20 to 79 years) in South Africa was estimated to be 4.3% in 2010 and is expected to rise to 4.9% by 2030 [2]. The disease has also contributed considerably to mortality - according to *Statistics South Africa,* diabetes caused 3.3% of the deaths recorded in 2008 [3]. The delivery of appropriate care for patients living with diabetes is thus a major challenge within South Africa’s health system.

In 2011, the South African National Department of Health drafted a National Health Insurance (NHI) Policy “Green” Paper in an effort to establish a more effective, and equitable, health system. A risk-adjusted capitation approach was proposed as a possible method of reimbursing accredited providers within the NHI dispensation [4].

Capitation is a form of funding in which health service providers are paid an agreed upon fixed premium by a health fund in advance of services delivered to members of that fund for a specified period [5]. The use of capitation is wide-spread; elements of risk-adjusted capitation can, for example, be found in the United Kingdom’s National Health Service, France’s *Assurance-maladie* and Israel’s National Health Insurance Law [6, 7]. More recently, capitation has been employed as a payment option in Ghana’s National Health Insurance Scheme [8].

Although the capitation approach creates an obvious incentive for service providers to deliver care in a cost effective manner, practicalities relating to the implementation of a capitation system need to be carefully addressed. A study of capitated payments relating to the management of patients with diabetes under Medicare in the United States, for example, found a risk of overpayment for the care of relatively healthy patients and a potential for under-treatment of those patients who were very ill, as the latter were more expensive to manage [9].

Against this background, yielding ‘best practice’ lessons from other sectors, including the private sector, with regards to implementing capitation systems would be useful. In the South African context, such lessons could be drawn from the Diabetes Management Programme (DMP) of the Centre for Diabetes and Endocrinology (CDE), a private sector healthcare provider. The DMP was launched in 1994 and has been described as “… a novel community-based capitation and risk-sharing model for diabetes management” [10].

Using a managed care approach, each patient enrolled in the DMP is offered a service package that adheres to ‘Minimum Care Guidelines.’ Medical aid schemes pay a monthly premium, in advance, for this care at a rate that is negotiated on an annual basis. However, the service providers are liable for any costs associated with diabetes-related emergencies (such as diabetic ketoacidosis) and are, thus, further incentivized to deliver a high quality of care. Whilst the clinical care offered through the DMP closely adheres to the guidelines of the International Diabetes Federation, distinctive features include the bundling of services within a capitation package as well as the inclusion of a risk-sharing/performance incentive element in the form of the diabetes emergency admission penalty [10].

The DMP has demonstrated positive clinical results; five-year outcome data showed a 40% overall reduction in the hospital admission rates for diagnoses that could be related to diabetes. Furthermore, 5-year HbA_1c_ levels showed a drop from 9.2% to 7.7% for patients with type 1 diabetes and from 8.8% to 7.4% for patients with type 2 diabetes [10].

Cost-effectiveness analysis assumes that decision-makers are seeking to maximize an effect within constrained resources and assesses whether alternative programmes are worth implementing by referring to an external standard [11]. Analyzing the cost-effectiveness of implementing an adaptation of this private sector clinical management programme to the public sector could, therefore, provide meaningful information, particularly in light of the NHI plan.

### Aim

The aim of the study was to assess whether a DMP capitation model, adapted for the South African public sector setting, was potentially cost-effective.

## Methods

**Figure 1:**
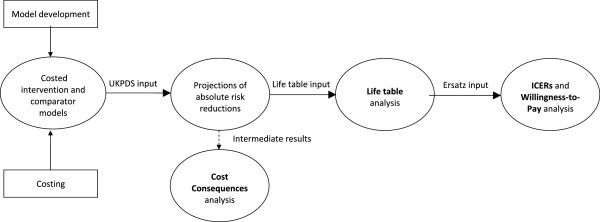
**Flow diagram of study methodology.**

The study was undertaken in the form of an economic evaluation that utilized probabilistic modelling. The analysis was undertaken for a government perspective, with particular relevance to the review of health service delivery and funding in the public sector. Ethical approval for the study was obtained from the Human Research Ethics Committee (Medical) of the University of the Witwatersrand.A flow diagram summarizing the study methodology is provided in Figure 1. The following key elements described in the figure are detailed below: (1) model development; (2) costing; (3) cost consequences analysis; (4) life table analysis; (5) incremental cost effectiveness ratios (ICERs) and Willingness-to-Pay (WTP) analysis.

### Model development

Secondary data used in the model design were obtained from published articles with the main source studies being Distiller et al. [10] and Klisiewicz and Raal [12] (the latter study sampled 150 patients attending public sector hospitals in Johannesburg, South Africa).

The ‘comparator’ was a usual practice clinical model based on the 2002 Society for Endocrinology, Metabolism and Diabetes of South Africa (SEMDSA) guidelines [13] adapted for the tertiary-level of care. The ‘intervention’ was the DMP capitation model adjusted for a primary health care (PHC) setting within the public service setting. This level of care was chosen because improving delivery of PHC services is a key focus of the NHI plan. Both models assumed that service users would be treated as outpatients.

Several assumptions were made in constructing the models. Firstly, the comparator model was set at the tertiary level of care because the studies referenced were undertaken in tertiary hospital settings. Furthermore, the 2002 SEMDSA guidelines were applied as written. This does not necessarily reflect the actual adherence to guidelines or the current form of practice (updated SEMDSA guidelines were, for example, published in 2012) [14]. Furthermore, only the management of type 2 DM was considered as literature related to type 2 DM in the South African context was readily available. Additionally, it was assumed that a substantially larger number of the patients utilizing PHC services for DM management would have this type of diabetes [15].

### Costing

Costing was calculated by following the convention of identifying, measuring and valuating relevant components [16]. Cost components in the models were identified using the SEDMSA guidelines for the comparator and DMP ‘Minimum Care Guidelines’ for the capitation intervention. This is summarized in Table 1. The main difference between the two models of care was the addition of training (based on the relevant CDE training course) and an incentive bonus (for every patient managed without a diabetes-related emergency) to the intervention model.

**Table 1:** **Model components and cost comparisons**

Cost category	Cost per capita (N = 150)			
	Intervention	Comparator	Difference	
**Direct costs**				
***1. Compensation***	ZAR 488.00	ZAR 432.00	**ZAR 56.00**	**+13.0%**
***2. Training***	ZAR 104.00*	ZAR 0.00	**ZAR 104.00**	**N/A**
***3. Investigations***	ZAR 850.62	ZAR 523.06	**ZAR 327.56**	**+62.6%**
***4. Medication & consumables***	ZAR 3 725.53	ZAR 3 223.41	**ZAR 502.12**	**+15.6%**
**Total direct costs**	ZAR 5 168.15	ZAR 4 178.47	**ZAR 989.68**	**+23.7%**
**Indirect costs**				
***5. Overheads***	ZAR 782.00	ZAR 768.00	**ZAR 14.00**	**+1.8%**
**Total costs**	ZAR 5 950.15	ZAR 4 946.47	**ZAR 1 003.68**	**+20.3%**
	(USD 724.74)	(USD 602.49)	**(USD 122.25)**	

*Combined training cost (based on the CDE’s 2013 fee structure) for one doctor and one nurse per patient seen.

Costs were measured for two consultations per annum, with relevant examinations and investigations counted for each visit. Doctors were understood to be internal medicine specialists in the comparator model and general medical practitioners in the capitation intervention model. Furthermore, it was assumed that for the group of 150 patients at least one medical practitioner and one nurse practitioner would be trained. An incentive bonus was also applied at an amount equivalent to the training cost per patient seen.

Secondary data of monthly medication usage were obtained from the CDE as well as the grey literature based on research undertaken at the University of the Witwatersrand [17]. Costing of medication was undertaken for diabetes medicines only; medication costs associated with the treatment of co-morbidities were not included. Reference was also made to the national *Essential Drugs Lists and Standard Treatment Guidelines* [18]. Consumable costs were based on an assumed monthly usage of a hundred component units (glucose strips and lancets) per patient for self-administered blood glucose tests as well as syringes and needles for insulin doses (for insulin users).

Valuations were based on cost information from the *Uniform Patient Fee Schedule* (UPFS) [19, 20] and the National Health Laboratory Service (NHLS) [21], as well as data sources of the CDE and the *Priority Cost Effective Lessons for Systems Strengthening South Africa* (PRICELESS SA) initiative (http://www.pricelesssa.ac.za). Cost valuations were mainly for 2012, and discounting was not used because the intervention and associated costs and health impacts were modeled for one year only. It was assumed that values would change minimally within this period so there was no inflation/deflation to a common year. For reporting purposes, the South African rand (ZAR) to United States dollar (USD) average exchange rate for 2012 was used. Exchange rate and gross domestic product (GDP) per capita data were obtained from the World Bank database (http://data.worldbank.org).

### Cost consequences analysis

Using the clinical information given for both the comparator and capitation intervention group, we estimated 10-year risks of cardiovascular mortality using the United Kingdom Prospective Diabetes Study (UKPDS) risk engine [22]. Inputs for the risk engine included mean age, ethnicity, smoking status, diabetes duration, HbA_1c_, systolic blood pressure, total cholesterol and high-density lipoprotein blood levels. A description, and explanatory details, of the UKPDS prediction equations can be found in an article written by Stevens et al. [23].

Health outcomes for all of the engine inputs, except diabetes duration, were obtained from the main source papers [10, 12]. The estimate for diabetes duration was taken from an alternative study undertaken on a patient population similar to that of the comparator study [24]. Final CVD risks were weighted for gender, ethnicity and smoking status. Cost consequences were then calculated from the UKPDS outputs for the intervention and comparator models, and worked out as a cost per 1% reduction in absolute risk. These were presented as intermediate results.

### Life table analysis

As part of economic evaluation, a multi-state life table is often employed to describe the differential morbidity and mortality experiences of a population under alternative intervention options [25, 26]. In this instance, two life tables were used - one following the mortality experience in a reference population (who experience the current mortality of people with diabetes in South Africa) and the other a population with diabetes who are exposed to the capitation intervention model.

Increases in cardiovascular disease (CVD) mortality as a result of having diabetes were modeled. The mortality rates in the South African population, as well as the mortality rates due to ischaemic heart disease (IHD) and stroke, were obtained from the South African National Burden of Disease study [27]. Within a standard life table, we separated CVD mortality from mortality due to other causes:


Where *M*_*T*_ = total mortality in age/sex group

*M*_*CVD*_ = mortality due to CVD

*M*_*other*_ = mortality due to all other causes

The relative risks of IHD and stroke mortality in people with diabetes from the Asia Pacific Cohort Studies Collaboration (APCSC) were applied to these mortality rates to estimate the mortality due to CVD in people with diabetes in the life table. The APCSC was used because it involved a meta-analysis of several longitudinal studies and hence provided a robust evidence base on which to calculate CVD mortality rates [28]. The number of people with diabetes was taken from an analysis of the non-fatal burden of disease attributed to diabetes in South Africa [29].


Where: *M*_*CVD*,*DM*_ = Mortality rate due to CVD in population with diabetes

*M*_*CVD*_ = Mortality rate due to CVD in population

*RRM*_*CVD,DM*_ = Relative risk of mortality due to CHD in population with diabetes

A ratio between the comparator and capitation intervention groups was used to calculate the relative reduction in mortality due to CVD expected as a result of the intervention. These reductions were applied to the CVD mortality rates in the life table and comparisons were made; comparisons included numbers of individuals surviving, cumulative years lived and life expectancy. Age categories in the life tables commenced from the 25–34 year group because inclusion of younger age groups resulted in a substantial over-estimation of years of life gained.


Where: *RR*_*m*_ = mortality risk reduction

*M*_*i*_ = mortality risk in intervention group

*M*_*c*_ = mortality risk in comparator group

Life tables were developed to estimate the impact of the capitation intervention on life expectancy and number of life years gained. A life table is generally used to estimate the mortality experience of a population and to calculate life expectancy at birth [30]. A life table calculates life expectancy using the formula [31]:


Where: *e*_*x*_ = life expectancy at age x

*T*_*x*_ = cumulative person years lived after age X

*l*_*x*_ = individuals alive at beginning of age x

A difference in cumulative person years lived after age 25 between the intervention and comparator populations were used in the ICER analysis.

#### ICER and WTP analysis

ICER analysis was undertaken by comparing the costs of the models to the final effect measure of life years gained (LYG), with life table outputs being used to derive the latter. ICERs were calculated by employing the conventional formula [32]:


Where: *I*_cost_ = cost of the intervention (that is, the adapted DMP model)

*C*_*cost*_ = cost of the diabetes management (public sector) comparator

*I*_effect_ = LYG from the comparator

*C*_effect_ = LYG from the comparator

Probabilistic sensitivity analysis was undertaken using a boot-strapping process. A probabilistic approach was adopted because of the level of uncertainty in the modelling. Indeed, probabilistic sensitivity analysis has been described as a more robust way of dealing with parameter uncertainty as compared to standard sensitivity analytical approaches [11]. Uncertainty values being included for fatal CHD and strokes parameters, as well as for the per capita costs of the models (as shown in Table 2).

**Table 2:** **Uncertainty parameters used in sensitivity analysis**

Data source	Parameter	Mean value	Lower and upper uncertainty values (***Ersatz***output)	
APCSC study [28]	Hazard ratio of fatal CHD in males	2.03%	-0.11%	4.41%
	Hazard ratio of fatal CHD in females	2.54%	-0.85%	6.42%
	Hazard ratio of fatal stroke in males	2.00%	-1.90%	5.13%
	Hazard ratio of fatal stroke in females	2.04%	-0.71%	5.04%
Intervention model (UKPDS risk engine output)	Absolute risk of fatal CHD in males	6.06%	3.92%	8.50%
	Absolute risk of fatal CHD in females	3.28%	2.21%	4.27%
	Absolute risk of fatal stroke in males	0.83%	0.76%	0.92%
	Absolute risk of fatal stroke in females	0.62%	0.54%	0.71%
Comparator model (UKPDS risk engine output)	Absolute risk of fatal CHD in males	8.74%	5.57%	11.66%
	Absolute risk of fatal CHD in females	4.78%	3.02%	6.41%
	Absolute risk of fatal stroke in males	1.13%	0.94%	1.29%
	Absolute risk of fatal stroke in females	0.82%	0.73%	0.94%
Intervention model (costing)	Per capita cost	ZAR 5950	ZAR 5401	ZAR 6533
Comparator model (costing)	Per capita cost	ZAR 4946	ZAR 4467	ZAR 5392

Modelling also allowed for Willingness-to-Pay (WTP) analysis. The WHO classifies a ‘highly cost-effective’ intervention as one that costs less than the national GDP per capita value [33]. Using this as definition, the outputs of the bootstrapping process were used to determine if the intervention fell below the WTP threshold.

### Software and computing

*Microsoft Excel*® was the main software application utilized for data capture and analysis. *Ersatz* (http://www.epigear.com), a boot-strap software add-in for Excel, was also used for uncertainty analysis.

## Results

The results as described below in terms of: (1) the costs of the models; (2) the cost consequences related to CVD risk reduction (intermediate results); (3) the life table modelling and, finally, (4) the ICER and WTP results.

### Costs of models

The annual per capita cost of intervention model was ZAR 5950 (USD 725) while that of the comparator was ZAR 4946 (USD 602), representing a cost increase of 20.3%. More detailed costs are displayed in Table 3. Apart from the obvious discrepancy of training costs, the greatest differences observed are in the categories of medication and consumables, and investigations.

**Table 3:** **Cardiovascular disease risk reduction and cost consequences**

	10- Year risk: Mean % (Standard Error)		Absolute risk reduction	Cost consequence
	***Intervention***	***Comparator***	***Percentage***	***Per 1***% ***risk reduction***
**Coronary Heart Disease (CHD)**				
**Males**	9.92 (0.93)	13.05 (1.23)	3.13	ZAR 320.97
**Females**	5.36 (0.49)	7.11 (0.70)	1.76	ZAR 571.70
**Fatal CHD**				
**Males**	6.06 (0.60)	8.74 (0.96)	2.68	ZAR 374.01
**Females**	3.28 (0.32)	4.78 (0.55)	1.50	ZAR 667.19
**Stroke**				
**Males**	6.40 (0.21)	7.12 (0.26)	0.72	ZAR 1397.88
**Females**	4.54 (0.13)	5.05 (0.20)	0.51	ZAR 1960.31
**Fatal Stroke**				
**Males**	0.83 (0.03)	1.13 (0.05)	0.30	ZAR 3345.60
**Females**	0.62 (0.03)	0.82 (0.03)	0.20	ZAR 5018.40

### Cost consequences results

The 10-year absolute risk reductions of CVDs potentially offered by the capitation intervention were projected by using the UKDPS risk engine (Table 4). The specific CVD outcomes analyzed by the engine are: coronary heart disease (CHD), fatal CHD, stroke and fatal stroke. The projected cost consequences per 1% absolute reduction in risk of the individual outcomes are reported as intermediary results. Costs associated with reductions in CHD (fatal and non-fatal) were substantially lower than those associated with stroke. The cost consequences of a 1% absolute risk reduction for fatal CHD, for example, was ZAR 374 (USD 46) and ZAR 667 (USD 81) for males and females respectively, compared to ZAR 3346 (USD 408) for males and ZAR 5018 (USD 611) for females for fatal stroke.

**Table 4:** **Summary of life table results**

Males							
Age (X)	Reported Population	Individuals with diabetes			Individuals on intervention		
A – Individuals surviving							
B – Cumulative years lived		A	B	C	A	B	C
C – Remaining life expectancy at age X							
**25-34**	37654	37 654	1515469	40.2	37654	1541953	41.0
**35-44**	116478	36837	1143011	31.0	36846	1169453	31.7
**45-54**	176800	34930	784172	22.4	34986	810293	23.2
**55-64**	170466	31632	451359	14.3	31889	475917	14.9
**65-74**	115191	23962	173390	7.2	24859	192175	7.7
**75+**	57486	7685	15155	2.0	9357	21094	2.3
**Females**							
**Age (X)**	**Reported Population**	**Individuals with diabetes**			**Individuals on intervention**		
**A – Individuals surviving**							
**B – Cumulative years lived**		**A**	**B**	**C**	**A**	**B**	**C**
**C – Remaining life expectancy at age X**							
**25-34**	70088	70088	3201030	45.7	70088	3273923	46.7
**35-44**	208972	68531	2507937	36.6	68544	2580762	37.7
**45-54**	284081	66330	1833631	27.6	66400	1906039	28.7
**55-64**	242488	63398	1184986	18.7	63693	1255573	19.7
**65-74**	173947	55288	591552	10.7	56402	655097	11.6
**75+**	114595	33421	148007	4.4	36533	190422	5.2

### Life table results

Summary results of the life tables for males and females are displayed in Table 4. As shown in the table, the intervention yielded a gain of life years and an increase in life expectancy in all categories (particularly the 45 – 64 year age bands). Furthermore, a greater benefit was seen in females as compared to males.

### ICER and WTP results

Probabilistic modelling was used for ICER analysis. This gave an ICER mean of ZAR 8 356 (USD 1018) per LYG (95% CI ZAR 2 794 - ZAR 14 811) by the capitation intervention. Furthermore, it was projected that a national programme based on the intervention would cost ZAR 792 million (USD 97 million) above the estimated baseline/comparator cost of ZAR 3.9 billion (USD 479 million), resulting in a total cost of ZAR 4.7 billion (USD 576 million), and would result in a mean cumulative gain of 96 997 years of life (95% CI 71 073 years – 113 994 years) in the population of people living with diabetes.

**Figure 2:**
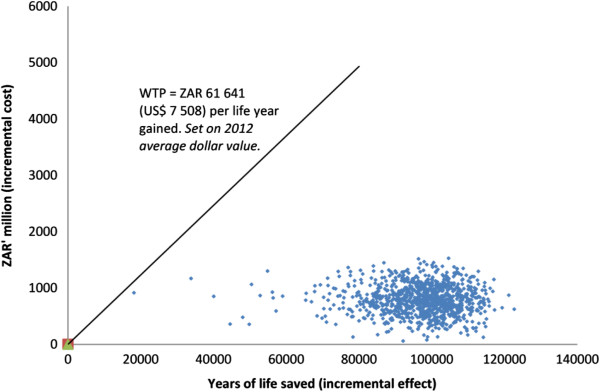
**Willingness-to-Pay analysis (using WHO standard** [33] **).**

The results of the WTP analysis are shown in Figure 2. Bootstrapping analysis (using 1000 iterations) showed all iterations of the intervention to be below South Africa’s 2012 GDP per capita and, hence, below the WTP threshold (in terms of the WHO’s “highly-cost effective” definition [33]).

## Discussion

The study demonstrates the potential utility of a DMP model, adapted for the PHC level, within the South African public sector. As shown in the modelling, all iterations of the intervention fell below the accepted WTP threshold. This finding, together with the life table results, indicates that the intervention could contribute to increasing life expectancy in South Africa (one of the strategic outputs of the *Negotiated Service Delivery Agreement* [34]) in a manner that is cost-effective.

Capitation models are used in the provision of health care in many countries [6]. Furthermore, implementing capitation systems has been employed as a component of health care reform. In New Zealand, for example, a strategic shift towards capitation formed an important part of the country’s Primary Care reforms [35], and in Thailand the use of a contract capitation model has been integral to its Social Health Insurance scheme [36].

Although the current NHI policy paper discusses the use of a risk-adjusted capitation model in the payment mechanism [4], implementing such a system in South Africa would present considerable challenges. Indeed, in reflecting on the first eighteen months of NHI development it has been acknowledged that the envisioned methods of provider payment “introduce considerable complexity into the negotiation … and it will take time to implement payment reforms” [37].

This will, undoubtedly, be compounded by the concomitant, and urgent, need to reduce the growing non-communicable disease burden. Against this background, the pertinence of the 2011 *South African Declaration on the Prevention of Non-Communicable Diseases* commitment to “[r]educing costs and (increasing) the efficiency of health interventions” can be appreciated [38]. The capitation intervention model analyzed in this paper may be of some assistance in fulfilling this commitment, at least with respect to the care of patients with diabetes in the public sector.

In addition to its potential cost-effectiveness, the capitation intervention could also contribute to a broader improvement in the quality of patient care. The DMP has been described as a managed care model driven, at least in part, by a desire to produce good outcomes for patients [10]. This is reflected in a commitment to individual professional development as well as the fostering of effective clinical teams. Enhancement of motivation, team dynamics and staff morale could thus be considered as key features of the model; it can perhaps be viewed as a holistic programme with the capitation element representing only one facet. Whilst these factors may be economically intangible, they are no less important particularly in light of the proposed performance-based mechanism in the NHI plan [4] and may, in fact, be essential to the model’s effective implementation in the public sector.

Challenges around adapting the CDE penalty system also merit discussion. One of the presumed strengths of the DMP model, as currently practised, is the inclusion of a penalty for diabetes emergencies. However, the application of such a disincentive in the public service setting is likely to be untenable as services in this sector are, by definition, not profit driven. For this reason, an incentive bonus per consultation for every patient successfully managed without a diabetes-related emergency was used as a proxy in this study. While the inclusion of such an incentive bonus may be debatable, and the actual type of incentive could vary, it is not without precedent [39]. The *Practice Incentives Program* of the Department of Human Services of the Australian Government, for example, appears to be based on a comparable incentive approach [40].

### Limitations

There are several limitations that apply to this study. Firstly, while it would have been preferable to use a comparator model that was at the same level of care as that of the intervention (that is, a primary care comparator) it was difficult to obtain relevant data to do so because of the paucity of relevant studies in the South African context. Furthermore, the intention was to develop the intervention and comparator as closely as possible to the actual service models used in the respective studies. Modelling primary health centre care as a comparator would have introduced additional uncertainty and required further assumptions.

Secondly, it was difficult to find a public sector equivalent for all of the cost components of the DMP. The cost, for example, of a dedicated 24-hour diabetes-related emergency line was not included as it was assumed that service users in the public sector would make use of general emergency medical services.

Thirdly, cost measurement relied largely on the UPFS and NHLS pricelists. Whilst there are obvious limitations in applying such generic guidelines in the estimation of costs, the scarcity of costing information relating to diabetes in the South African context made it difficult to find alternatives. Furthermore, these guidelines are used in actual practice. The UPFS, for example, provides a national standard for the billing of patients accessing provincial health facilities and guides medical schemes in reimbursing these facilities following the provision of services to their beneficiaries [41].

Fourthly, the use of LYG as an outcome measure constrained the study to mortality/fatality analysis. This was a limitation given the substantial morbidity arising from the microvascular complications of diabetes (e.g. renal failure, diabetic retinopathy) in South Africa [42]. While LYG did provide for ICER analysis relevant to policy and decision making, the inclusion of modelling around microvascular complications could be a valuable complement to this analysis.

Fifthly, there were limitations resulting from the use of the UKPDS risk engine. In addition to the obvious difficulty of applying a United Kingdom-based model to the South African setting, it is also probable that the treatment of diabetes, and its co-morbidities, has improved substantially since the engine was developed (the intervention trial on which the engine is based was completed in 1997 and the post-trial programme ended in 2007) [43]. However, while the risk engine has, indeed, been found to overestimate true risk, this has been seen as more of a limitation with regards to individual prognostication [44]. Moreover, the engine has been deemed to be a suitable tool for resource prioritization and allocation analysis [44, 45].

## Conclusions

The growing burden of diabetes represents a major public health challenge. Along with its resultant morbidity and mortality, the disease imposes a massive economic cost. Indeed, a recent report found that the global economic cost of diabetes is expected to rise from USD 500 billion in 2010 to approximately USD 745 billion by 2030, with low- and middle-income countries having to carry a substantial amount of the burden [46]. Given the resource constraints existent in South Africa, the identification of potential ‘best buy’ models of diabetes care is an imperative.

Preliminary analysis using probabilistic modelling found the capitation intervention model of type 2 diabetes care to be cost-effective. However, given the difficulty in adapting the DMP to the PHC level in the South African public sector context, further prospective research is needed.

A public service-based pilot study could, for example, allow for the collection of primary data and, consequently, more robust economic analysis. A pilot study could also yield information relevant to health services planning, including human resource, financial and logistical requirements. This could, in turn, inform to the final design of an implementable, adapted service model. Referral pathways from such a service to specialist levels of care, where appropriate, could also be delineated. Furthermore, prospective studies could include additional qualitative components that examine how other factors, such as staff motivation and multi-disciplinary team dynamics, influence patient care and health outcomes. Such research could contribute to an improved, cost-effective model of care for people with type 2 diabetes within the NHI dispensation.

## Abbreviations

CDE: Centre for Diabetes and Endocrinology
CHD: Coronary heart disease
CI: Confidence interval
CVD: Cardiovascular disease
DMP: Diabetes Management Programme
GDP: Gross Domestic Product
HbA_1c_: Glycosylated haemoglobin
ICER: Incremental cost-effectiveness ratio
IHD: Ischaemic heart disease
LYG: Life year gained
PHC: Primary Health Care
PRICELESS SA: Priority Cost Effective Lessons for Systems Strengthening South Africa
NHI: National Health Insurance
NHLS: National Health Laboratory Service
UKPDS: United Kingdom Prospective Diabetes Study
UPFS: Uniform patient fee schedule
USD: United States dollar
WTP: Willingness-to-pay
ZAR: South African rand.
